# (4-Chlorophenyl)(2-hydroxy-7-methoxynaphthalen-1-yl)methanone

**DOI:** 10.1107/S1600536808039603

**Published:** 2008-11-29

**Authors:** Ryosuke Mitsui, Kosuke Nakaema, Keiichi Noguchi, Noriyuki Yonezawa

**Affiliations:** a Department of Organic and Polymer Materials Chemistry, Tokyo University of Agriculture & Technology, 2-24-16 Naka-machi, Koganei, Tokyo 184-8588, Japan; b Instrumentation Analysis Center, Tokyo University of Agriculture & Technology, 2-24-16 Naka-machi, Koganei, Tokyo 184-8588, Japan

## Abstract

The title compound, C_18_H_13_ClO_3_, has an intra­molecular O—H⋯O=C hydrogen bond between the carbonyl group and the hydr­oxy substituent on the naphthalene ring system. The angle between the C=O bond plane and the naphthalene ring system is relatively small [20.96 (8)°]. The angle between the benzene ring and the carbonyl group is rather large [35.65 (9)°] compared to that in an analogous compound [3.43 (11)°] having a meth­oxy group instead of a hydroxy substituent.

## Related literature

For the structures of closely related compounds, see: Nakaema *et al.* (2007 ▶, 2008 ▶); Mitsui *et al.* (2008 ▶).
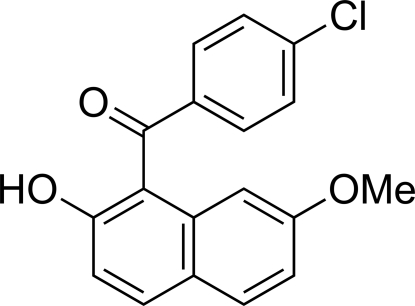


## Experimental

### 

#### Crystal data

C_18_H_13_ClO_3_
*M* *_r_* = 312.73
Orthorhombic, 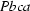

*a* = 17.8030 (3) Å
*b* = 8.68121 (10) Å
*c* = 18.8683 (3) Å
*V* = 2916.14 (8) Å^3^
*Z* = 8
Cu *K*α radiation
μ = 2.41 mm^−1^
*T* = 123 K
0.60 × 0.15 × 0.05 mm

#### Data collection

Rigaku R-AXIS RAPID diffractometer
Absorption correction: numerical (*NUMABS*; Higashi, 1999 ▶) *T*_min_ = 0.485, *T*_max_ = 0.886
49864 measured reflections
2669 independent reflections
2347 reflections with *I* > 2σ(*I*)
*R*_int_ = 0.033

#### Refinement

*R*[*F* ^2^ > 2σ(*F* ^2^)] = 0.033
*wR*(*F* ^2^) = 0.096
*S* = 1.08
2669 reflections
205 parameters
H atoms treated by a mixture of independent and constrained refinement
Δρ_max_ = 0.17 e Å^−3^
Δρ_min_ = −0.25 e Å^−3^

### 

Data collection: *PROCESS-AUTO* (Rigaku, 1998 ▶); cell refinement: *PROCESS-AUTO*; data reduction: *CrystalStructure* (Rigaku/MSC, 2004 ▶); program(s) used to solve structure: *SIR2004* (Burla *et al.*, 2005 ▶); program(s) used to refine structure: *SHELXL97* (Sheldrick, 2008 ▶); molecular graphics: *ORTEPIII* (Burnett & Johnson, 1996 ▶); software used to prepare material for publication: *SHELXL97*.

## Supplementary Material

Crystal structure: contains datablocks global, I. DOI: 10.1107/S1600536808039603/su2069sup1.cif

Structure factors: contains datablocks I. DOI: 10.1107/S1600536808039603/su2069Isup2.hkl

Additional supplementary materials:  crystallographic information; 3D view; checkCIF report

## Figures and Tables

**Table 1:** Hydrogen-bond geometry (Å, °)

*D*—H⋯*A*	*D*—H	H⋯*A*	*D*⋯*A*	*D*—H⋯*A*
O2—H2⋯O1	0.94 (2)	1.71 (2)	2.5573 (16)	148 (2)

**Fig. 1.**
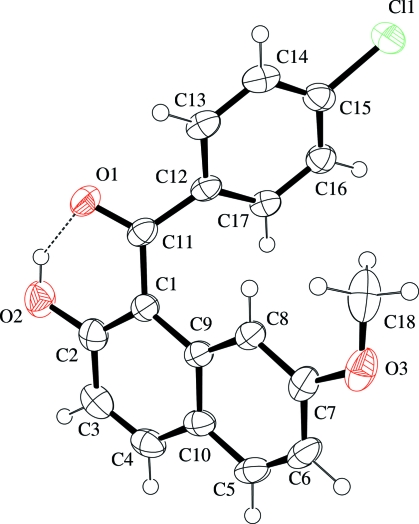
The molecular structure of compound (II), showing 50% probability displacement ellipsoids. The intramolecular hydrogen bond is shown as a dashed line.

**Fig. 2.**
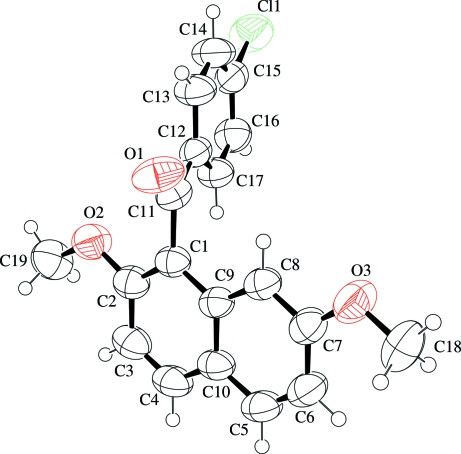
The molecular structure of compound (I) [Mitsui et al., 2008], showing 50% probability displacement ellipsoids.

**Fig. 3.**
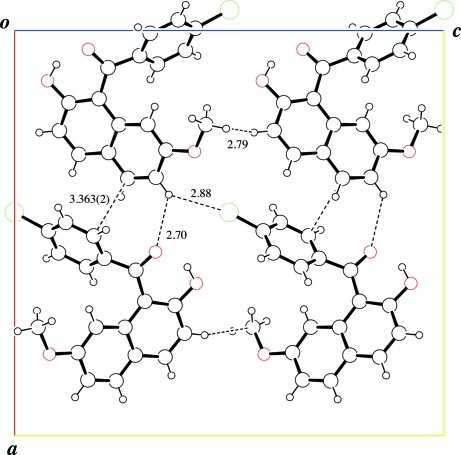
A partial crystal packing diagram of compound (II), viewed down the b-axis (the intermolecular C—H···O and C—H···π interactions are shown as dashed lines).

**Fig. 4.**
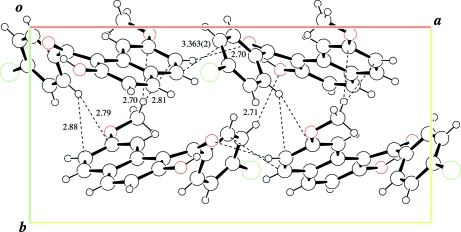
A partial crystal packing diagram of compound (II), viewed down the c-axis (the intermolecular C—H···O and C—H···π interactions are shown as dashed lines).

## supplementary crystallographic information

### Comment

Recently, we have reported on the crystal structure of 1-(4-chlorobenzoyl)-2,7-dimethoxynaphthalene, (I) [Mitsui *et al.*, 2008]. As a part of our ongoing studies on the synthesis and crystal structure analyses of aroylated naphthalene derivatives, we prepared and analysed the crystal structure of the title compound, (II). Compound (II) was prepared by regioselective demethylation reaction of compound (I) with aluminium trichloride.

The molecular structure of compound (II) is illustrated in Fig. 1. In analogous aroylated naphthalenes, for example compound (I) shown in Fig. 2, the C═O bond plane is almost perpendicular to the mean plane of the naphthalene ring (Nakaema *et al.*, 2007, 2008; Mitsui *et al.*, 2008). In contrast, the angle between the C═O bond and the naphthalene ring in compound (II) is considerably smaller, i.e. 20.96 (8)°. This is apparently caused by the intramolecular O—H···O═C hydrogen bond, which forms a six-membered ring including the carbonyl group and an edge of the naphthalene ring (Fig. 1 and Table 1).

In compound (I) the C═O bond and the benzene ring are almost coplanar with a dihedral angle of 3.43 (11)°. In compound (II) the mean plane of the benzene ring is twisted away from the C═O bond by 35.65 (9)°. This is presumably caused by the release of the rather large steric repulsion between the benzene ring and the naphthalene ring brought about by the small angle of the C═O bond plane and the naphthalene ring. The dihedral angle between the naphthalene ring (C1—C10) and the benzene ring (C12—C17) is 58.10 (6)°.

In the crystal structure the molecular packing of (II) is mainly stabilized by van der Waals interactions. The naphthalene rings interact with the phenyl rings [C5···C13 = 3.363 (2) Å] and the carbonyl groups [H6···O1 = 2.70 Å] along the *a*-axis. They also interact with the methyl groups [H3···C18 = 2.79 Å] and aroyl groups [H6···Cl1 = 2.88 Å] along the *c*-axis (Fig. 3). On the other hand, the naphthalene rings also interact with the methyl groups [C6···H18B = 2.81 Å, C7···H18B = 2.70 Å] and the phenyl rings [C6···H17 = 2.88 Å, C7···H17 = 2.79 Å] along the *b*-axis. The naphthalene rings are almost perpendicular to the phenyl rings of the adjacent molecules along the *b*-axis. In addition, the hydroxy groups interact with the phenyl rings [O2···H14 = 2.71 Å] along the *b*-axis (Fig. 4).

### Experimental

To a solution of 1-(4-chlorobenzoyl)-2,7-dimethoxynaphthalene (33 mg, 0.10 mmol) in CH_2_Cl_2_ (1.0 ml) was added AlCl_3_ (67 mg, 0.50 mmol). The reaction mixture was refluxed for 30 min giving a dark red solution, which was then poured into H_2_O (5 ml) and CHCl_3_ (3 ml). The aqueous layer was extracted with CHCl_3_ (3 × 5 ml). The combined organic layers were washed with brine (3 × 10 ml), and dried over MgSO_4_ overnight. The solvent was removed *in vacuo* and the crude material was purified by recrystallization from hexane to give compound (II) as yellow platelets (m.p. 391–391.5 K, yield 23 mg, 75%).

Spectroscopic Data: ^1^H NMR (300 MHz, CDCl_3_) δ 11.35 (s, 1H), 7.85 (d, 1H), 7.63 (d, 1H), 7.58 (d, 2H), 7.40 (d, 2H), 7.07 (d, 1H), 6.91 (dd, 1H), 6.58 (d, 1H), 3.37 (s, 3H); ^13^C NMR (75 MHz, CDCl_3_) δ 199.1, 162.6, 158.2, 138.8, 138.7, 136.5, 133.8, 130.7, 130.2, 128.9, 123.7, 116.4, 115.8, 113.4, 106.5, 54.5; IR (KBr): 3434, 1623, 1583, 1513, 1214, 843.

Anal. Calcd for C_18_H_13_ClO_3_: C 69.13, H 4.19. Found: C 69.11, H 4.09.

### Refinement

All the H-atoms could be located in difference Fourier maps. The OH hydrogen atom was freely refined: O2—H2 = 0.94 (2) Å. The C-bound H-atoms were subsequently refined as riding atoms, with C—H = 0.95 (aromatic) and 0.98 (methyl) Å, and *U*_iso_(H) = 1.2*U*_eq_(C).

### Crystal data

**Table:**

C_18_H_13_ClO_3_	*D*_x_ = 1.425 Mg m^−^^3^
*M**_r_* = 312.73	Melting point = 391.0–391.5 K
Orthorhombic, *P**b**c**a*	Cu *K*α radiation λ = 1.54187 Å
Hall symbol: -P 2ac 2ab	Cell parameters from 42602 reflections
*a* = 17.8030 (3) Å	θ = 3.4–68.2º
*b* = 8.68121 (10) Å	µ = 2.41 mm^−^^1^
*c* = 18.8683 (3) Å	*T* = 123 K
*V* = 2916.14 (8) Å^3^	Plate, yellow
*Z* = 8	0.60 × 0.15 × 0.05 mm
*F*_000_ = 1296	

### Data collection

**Table:**

Rigaku R-AXIS RAPID diffractometer	2669 independent reflections
Radiation source: rotating anode	2347 reflections with *I* > 2σ(*I*)
Monochromator: graphite	*R*_int_ = 0.033
Detector resolution: 10.00 pixels mm^-1^	θ_max_ = 68.2º
*T* = 123 K	θ_min_ = 4.7º
ω scans	*h* = −21→21
Absorption correction: numerical(NUMABS; Higashi, 1999)	*k* = −10→10
*T*_min_ = 0.485, *T*_max_ = 0.886	*l* = −22→22
49864 measured reflections	

### Refinement

**Table:**

Refinement on *F*^2^	Hydrogen site location: dfimap
Least-squares matrix: full	H atoms treated by a mixture of independent and constrained refinement
*R*[*F*^2^ > 2σ(*F*^2^)] = 0.033	*w* = 1/[σ^2^(*F*_o_^2^) + (0.0544*P*)^2^ + 0.6658*P*] where *P* = (*F*_o_^2^ + 2*F*_c_^2^)/3
*wR*(*F*^2^) = 0.096	(Δ/σ)_max_ = 0.001
*S* = 1.08	Δρ_max_ = 0.17 e Å^−^^3^
2669 reflections	Δρ_min_ = −0.25 e Å^−^^3^
205 parameters	Extinction correction: SHELXL97 (Sheldrick, 2008), Fc^*^=kFc[1+0.001xFc^2^λ^3^/sin(2θ)]^-1/4^
Primary atom site location: structure-invariant direct methods	Extinction coefficient: 0.00062 (11)
Secondary atom site location: difference Fourier map	

### Special details

**Table:**

Geometry. All e.s.d.'s (except the e.s.d. in the dihedral angle between two l.s. planes) are estimated using the full covariance matrix. The cell e.s.d.'s are taken into account individually in the estimation of e.s.d.'s in distances, angles and torsion angles; correlations between e.s.d.'s in cell parameters are only used when they are defined by crystal symmetry. An approximate (isotropic) treatment of cell e.s.d.'s is used for estimating e.s.d.'s involving l.s. planes.
Refinement. Refinement of *F*^2^ against ALL reflections. The weighted *R*-factor *wR* and goodness of fit *S* are based on *F*^2^, conventional *R*-factors *R* are based on *F*, with *F* set to zero for negative *F*^2^. The threshold expression of *F*^2^ > σ(*F*^2^) is used only for calculating *R*-factors(gt) *etc*. and is not relevant to the choice of reflections for refinement. *R*-factors based on *F*^2^ are statistically about twice as large as those based on *F*, and *R*- factors based on ALL data will be even larger.

### Fractional atomic coordinates and isotropic or equivalent isotropic displacement parameters (Å^2^)

**Table:**

	*x*	*y*	*z*	*U*_iso_*/*U*_eq_	
Cl1	−0.05110 (2)	0.23154 (6)	0.500766 (19)	0.05295 (17)	
O1	0.04658 (5)	0.08073 (14)	0.17181 (6)	0.0439 (3)	
O2	0.12423 (7)	0.21814 (15)	0.07692 (6)	0.0485 (3)	
H2	0.0840 (13)	0.163 (3)	0.0974 (11)	0.070 (7)*	
O3	0.29872 (6)	0.03670 (13)	0.41875 (6)	0.0474 (3)	
C1	0.16824 (8)	0.17285 (16)	0.19660 (7)	0.0299 (3)	
C2	0.17846 (9)	0.22425 (17)	0.12659 (8)	0.0373 (3)	
C3	0.24694 (10)	0.28979 (18)	0.10428 (8)	0.0432 (4)	
H3	0.2518	0.3289	0.0575	0.052*	
C4	0.30589 (9)	0.29694 (18)	0.14974 (9)	0.0415 (4)	
H4	0.3514	0.3439	0.1347	0.050*	
C5	0.36473 (8)	0.23357 (18)	0.26380 (10)	0.0419 (4)	
H5	0.4104	0.2779	0.2476	0.050*	
C6	0.36219 (8)	0.16956 (18)	0.32922 (9)	0.0433 (4)	
H6	0.4052	0.1714	0.3590	0.052*	
C7	0.29510 (8)	0.10024 (17)	0.35260 (8)	0.0368 (3)	
C8	0.23210 (7)	0.09796 (16)	0.31021 (7)	0.0312 (3)	
H8	0.1880	0.0473	0.3262	0.037*	
C9	0.23251 (7)	0.17038 (15)	0.24311 (7)	0.0293 (3)	
C10	0.30113 (7)	0.23607 (16)	0.21894 (9)	0.0341 (3)	
C11	0.09175 (7)	0.12732 (16)	0.21759 (8)	0.0325 (3)	
C12	0.06218 (7)	0.14707 (16)	0.29105 (8)	0.0304 (3)	
C13	0.00695 (8)	0.04620 (17)	0.31572 (8)	0.0354 (3)	
H13	−0.0079	−0.0389	0.2873	0.042*	
C14	−0.02637 (8)	0.06910 (18)	0.38113 (8)	0.0389 (4)	
H14	−0.0630	−0.0011	0.3984	0.047*	
C15	−0.00543 (8)	0.19623 (18)	0.42098 (8)	0.0364 (3)	
C16	0.04883 (8)	0.29823 (17)	0.39772 (8)	0.0346 (3)	
H16	0.0626	0.3846	0.4258	0.041*	
C17	0.08296 (8)	0.27253 (16)	0.33264 (8)	0.0320 (3)	
H17	0.1208	0.3411	0.3163	0.038*	
C18	0.23232 (12)	−0.0353 (2)	0.44536 (9)	0.0560 (5)	
H18A	0.2422	−0.0770	0.4927	0.067*	
H18B	0.2174	−0.1192	0.4135	0.067*	
H18C	0.1918	0.0407	0.4481	0.067*	

### Atomic displacement parameters (Å^2^)

**Table:**

	*U*^11^	*U*^22^	*U*^33^	*U*^12^	*U*^13^	*U*^23^
Cl1	0.0399 (3)	0.0813 (4)	0.0376 (2)	−0.0016 (2)	0.00670 (15)	0.00181 (19)
O1	0.0292 (6)	0.0561 (7)	0.0463 (6)	−0.0002 (5)	−0.0077 (4)	−0.0137 (5)
O2	0.0513 (7)	0.0577 (8)	0.0364 (6)	0.0083 (6)	−0.0084 (5)	−0.0005 (5)
O3	0.0542 (7)	0.0414 (6)	0.0466 (6)	0.0105 (5)	−0.0200 (5)	−0.0052 (5)
C1	0.0286 (7)	0.0260 (7)	0.0351 (7)	0.0025 (5)	−0.0004 (5)	−0.0043 (5)
C2	0.0416 (8)	0.0317 (8)	0.0385 (8)	0.0069 (6)	−0.0016 (6)	−0.0048 (6)
C3	0.0554 (9)	0.0328 (9)	0.0413 (8)	0.0008 (7)	0.0116 (8)	0.0006 (7)
C4	0.0395 (8)	0.0313 (8)	0.0536 (10)	−0.0038 (6)	0.0153 (7)	−0.0075 (7)
C5	0.0245 (7)	0.0363 (8)	0.0649 (10)	−0.0006 (6)	0.0027 (7)	−0.0195 (7)
C6	0.0268 (7)	0.0396 (9)	0.0635 (11)	0.0073 (6)	−0.0130 (7)	−0.0200 (8)
C7	0.0368 (8)	0.0295 (8)	0.0441 (8)	0.0104 (6)	−0.0112 (6)	−0.0111 (6)
C8	0.0274 (7)	0.0263 (7)	0.0398 (7)	0.0028 (5)	−0.0033 (5)	−0.0053 (6)
C9	0.0253 (7)	0.0237 (7)	0.0388 (7)	0.0025 (5)	0.0001 (5)	−0.0074 (5)
C10	0.0276 (7)	0.0267 (7)	0.0482 (9)	0.0006 (5)	0.0051 (6)	−0.0102 (6)
C11	0.0264 (7)	0.0290 (7)	0.0422 (8)	0.0029 (6)	−0.0056 (6)	−0.0043 (6)
C12	0.0213 (6)	0.0298 (7)	0.0402 (7)	0.0025 (5)	−0.0033 (5)	−0.0007 (6)
C13	0.0263 (7)	0.0305 (8)	0.0493 (8)	0.0003 (6)	−0.0043 (6)	−0.0018 (6)
C14	0.0274 (7)	0.0392 (9)	0.0502 (9)	−0.0027 (6)	−0.0010 (6)	0.0097 (7)
C15	0.0281 (7)	0.0448 (9)	0.0363 (7)	0.0057 (6)	0.0007 (6)	0.0059 (6)
C16	0.0312 (7)	0.0342 (8)	0.0383 (8)	0.0026 (6)	−0.0017 (6)	−0.0028 (6)
C17	0.0258 (7)	0.0298 (7)	0.0405 (8)	0.0001 (5)	−0.0008 (6)	0.0000 (6)
C18	0.0896 (14)	0.0381 (9)	0.0402 (9)	−0.0063 (9)	−0.0156 (9)	0.0008 (7)

### Geometric parameters (Å, °)

**Table:**

Cl1—C15	1.7381 (15)	C7—C8	1.3778 (19)
O1—C11	1.2476 (17)	C8—C9	1.414 (2)
O2—C2	1.3466 (19)	C8—H8	0.9500
O2—H2	0.94 (2)	C9—C10	1.4233 (19)
O3—C7	1.3661 (19)	C11—C12	1.493 (2)
O3—C18	1.429 (2)	C12—C17	1.392 (2)
C1—C2	1.406 (2)	C12—C13	1.397 (2)
C1—C9	1.4422 (19)	C13—C14	1.384 (2)
C1—C11	1.4722 (19)	C13—H13	0.9500
C2—C3	1.410 (2)	C14—C15	1.387 (2)
C3—C4	1.357 (2)	C14—H14	0.9500
C3—H3	0.9500	C15—C16	1.382 (2)
C4—C10	1.411 (2)	C16—C17	1.388 (2)
C4—H4	0.9500	C16—H16	0.9500
C5—C6	1.354 (3)	C17—H17	0.9500
C5—C10	1.414 (2)	C18—H18A	0.9800
C5—H5	0.9500	C18—H18B	0.9800
C6—C7	1.408 (2)	C18—H18C	0.9800
C6—H6	0.9500		
			
C2—O2—H2	106.3 (13)	C4—C10—C9	119.88 (14)
C7—O3—C18	117.31 (12)	C5—C10—C9	119.30 (15)
C2—C1—C9	118.28 (13)	O1—C11—C1	119.80 (13)
C2—C1—C11	117.23 (13)	O1—C11—C12	116.93 (12)
C9—C1—C11	124.48 (12)	C1—C11—C12	123.04 (12)
O2—C2—C1	123.27 (15)	C17—C12—C13	119.32 (13)
O2—C2—C3	115.35 (14)	C17—C12—C11	121.30 (13)
C1—C2—C3	121.36 (14)	C13—C12—C11	119.07 (13)
C4—C3—C2	119.91 (15)	C14—C13—C12	120.60 (14)
C4—C3—H3	120.0	C14—C13—H13	119.7
C2—C3—H3	120.0	C12—C13—H13	119.7
C3—C4—C10	121.42 (14)	C13—C14—C15	118.87 (14)
C3—C4—H4	119.3	C13—C14—H14	120.6
C10—C4—H4	119.3	C15—C14—H14	120.6
C6—C5—C10	121.68 (15)	C16—C15—C14	121.71 (14)
C6—C5—H5	119.2	C16—C15—Cl1	119.28 (12)
C10—C5—H5	119.2	C14—C15—Cl1	118.97 (12)
C5—C6—C7	119.28 (14)	C15—C16—C17	118.95 (14)
C5—C6—H6	120.4	C15—C16—H16	120.5
C7—C6—H6	120.4	C17—C16—H16	120.5
O3—C7—C8	124.26 (14)	C16—C17—C12	120.53 (13)
O3—C7—C6	114.75 (13)	C16—C17—H17	119.7
C8—C7—C6	120.98 (15)	C12—C17—H17	119.7
C7—C8—C9	120.61 (13)	O3—C18—H18A	109.5
C7—C8—H8	119.7	O3—C18—H18B	109.5
C9—C8—H8	119.7	H18A—C18—H18B	109.5
C8—C9—C10	118.02 (13)	O3—C18—H18C	109.5
C8—C9—C1	123.19 (12)	H18A—C18—H18C	109.5
C10—C9—C1	118.68 (13)	H18B—C18—H18C	109.5
C4—C10—C5	120.77 (14)		
			
C9—C1—C2—O2	173.72 (13)	C6—C5—C10—C9	0.3 (2)
C11—C1—C2—O2	−7.2 (2)	C8—C9—C10—C4	174.23 (12)
C9—C1—C2—C3	−7.8 (2)	C1—C9—C10—C4	−2.23 (19)
C11—C1—C2—C3	171.28 (13)	C8—C9—C10—C5	−3.14 (19)
O2—C2—C3—C4	−177.98 (14)	C1—C9—C10—C5	−179.59 (12)
C1—C2—C3—C4	3.4 (2)	C2—C1—C11—O1	26.3 (2)
C2—C3—C4—C10	1.8 (2)	C9—C1—C11—O1	−154.68 (14)
C10—C5—C6—C7	1.6 (2)	C2—C1—C11—C12	−148.00 (14)
C18—O3—C7—C8	−0.5 (2)	C9—C1—C11—C12	31.0 (2)
C18—O3—C7—C6	179.99 (13)	O1—C11—C12—C17	−139.55 (14)
C5—C6—C7—O3	178.89 (13)	C1—C11—C12—C17	34.9 (2)
C5—C6—C7—C8	−0.6 (2)	O1—C11—C12—C13	33.95 (19)
O3—C7—C8—C9	178.21 (12)	C1—C11—C12—C13	−151.57 (13)
C6—C7—C8—C9	−2.4 (2)	C17—C12—C13—C14	−0.7 (2)
C7—C8—C9—C10	4.17 (19)	C11—C12—C13—C14	−174.37 (13)
C7—C8—C9—C1	−179.55 (13)	C12—C13—C14—C15	1.7 (2)
C2—C1—C9—C8	−169.18 (13)	C13—C14—C15—C16	−1.4 (2)
C11—C1—C9—C8	11.8 (2)	C13—C14—C15—Cl1	176.26 (11)
C2—C1—C9—C10	7.07 (19)	C14—C15—C16—C17	0.2 (2)
C11—C1—C9—C10	−171.92 (13)	Cl1—C15—C16—C17	−177.50 (11)
C3—C4—C10—C5	175.05 (14)	C15—C16—C17—C12	0.8 (2)
C3—C4—C10—C9	−2.3 (2)	C13—C12—C17—C16	−0.5 (2)
C6—C5—C10—C4	−177.05 (14)	C11—C12—C17—C16	172.96 (13)

### Hydrogen-bond geometry (Å, °)

**Table:**

*D*—H···*A*	*D*—H	H···*A*	*D*···*A*	*D*—H···*A*
O2—H2···O1	0.94 (2)	1.71 (2)	2.5573 (16)	148 (2)
